# *Plasmodium vivax* Duffy Binding Protein-Based Vaccine: a Distant Dream

**DOI:** 10.3389/fcimb.2022.916702

**Published:** 2022-07-13

**Authors:** Sonalika Kar, Abhinav Sinha

**Affiliations:** Parasite Host Biology, Indian Council of Medical Research-National Institute of Malaria Research, New Delhi, India

**Keywords:** Malaria, *Plasmodium vivax*, vaccine, blood stage malaria antigen, *Pv*DBP

## Abstract

The neglected but highly prevalent *Plasmodium vivax* in South-east Asia and South America poses a great challenge, with regards to long-term in-vitro culturing and heavily limited functional assays. Such visible challenges as well as narrowed progress in development of experimental research tools hinders development of new drugs and vaccines. The leading vaccine candidate antigen *Plasmodium vivax* Duffy Binding Protein (*Pv*DBP), is essential for reticulocyte invasion by binding to its cognate receptor, the Duffy Antigen Receptor for Chemokines (DARC), on the host’s reticulocyte surface. Despite its highly polymorphic nature, the amino-terminal cysteine-rich region II of *Pv*DBP (*Pv*DBPII) has been considered as an attractive target for vaccine-mediated immunity and has successfully completed the clinical trial Phase 1. Although this molecule is an attractive vaccine candidate against vivax malaria, there is still a question on its viability due to recent findings, suggesting that there are still some aspects which needs to be looked into further. The highly polymorphic nature of *Pv*DBPII and strain-specific immunity due to *Pv*DBPII allelic variation in Bc epitopes may complicate vaccine efficacy. Emergence of various blood-stage antigens, such as *Pv*RBP, *Pv*EBP and supposedly many more might stand in the way of attaining full protection from *Pv*DBPII. As a result, there is an urgent need to assess and re-assess various caveats connected to *Pv*DBP, which might help in designing a long-term promising vaccine for *P. vivax* malaria. This review mainly deals with a bunch of rising concerns for validation of DBPII as a vaccine candidate antigen for *P. vivax* malaria.

## Preface

Infectious diseases have played an important role in modeling human demography and genetics. Malaria is considered to be one of the most devastating infectious diseases affecting mankind and is believed to be one of the strongest selective pressures in recent human history (Haldane, 2004; Kwiatkowski, 2005). At least nine species of the unicellular eukaryotic parasite of genus *Plasmodium* are reported to cause infection in humans including *P. falciparum*, *P. vivax*, *P. malariae*, *P. ovale curtisi*, *P. ovale wallikeri* (Sutherland et al., 2010), *P. knowlesi*, *P. cynomolgi* (Ta et al., 2014), *P. simium* (Deane, 1992; Brasil et al., 2017), and *P. brasilianum* (Lalremruata et al., 2015). Out of these nine species, only *Plasmodium falciparum* and *Plasmodium vivax* emerge to be the major threats escalating the malaria load globally. Though great progress has been made in the fight against malaria since 2000, an increase in the number of cases in the last few years has placed doubt on the objective of eliminating the illness. In 2020, an estimated 241 million malaria cases were reported in 85 malaria-endemic countries (World Health Organization, 2021). The WHO African Region accounted for around 95% of cases in 2020, with an anticipated 228 million cases (World Health Organization, 2021). There has been an increase in the proportion of malaria caused by *P. vivax* in co-endemic regions where intensive malaria-control measures have lowered the burden of *P. falciparum*. In co-endemic areas, there is an increased risk of *P. vivax* after *P. falciparum* therapy, suggesting that universal radical cure for both parasites might be beneficial in some situations.

*P. vivax* is by far the most predominant source of human malaria across Asia and the Asia-Pacific regions, which account for approximately 80% of the worldwide *P. vivax* burden due to large populations and a diminishing prevalence of *P. falciparum* infections (Howes et al., 2016). Its existence has also been reported in the horn of Africa, Madagascar, and parts of Central and South America (World Health Organization, 2020). The sensitivity of the present-generation RDTs employed for *P. vivax* diagnosis is comparable to that of microscopy (Chu and White, 2021). In malaria-endemic areas, it has been found that ultrasensitive PCR technologies detects parasite densities as low as 28/ml (Imwong et al., 2014). This indicates a substantially greater prevalence of asymptomatic *P. vivax* infection than previously thought. In areas where *P. falciparum* and *P. vivax* malaria coexist, the *P. vivax* burden has overtaken the *P. falciparum* burden (Battle et al., 2019). *P. falciparum*, which is well known to cause complicated and fatal malaria, has overshadowed the clinical and public health importance of *P. vivax* malaria (Baird, 2007; Conway, 2007). Contrary to the belief that *P. vivax* causes a relatively benign and self-limiting infection, evidence documenting severe and complicated *P. vivax* malaria are escalating gradually (Price et al., 2007; Herrera et al., 2007; Baird, 2013a; Baird, 2013b). Concomitant or chronic illness could result into a severe *P. vivax* infection.

It is increasingly becoming visible that efforts towards understanding *P. vivax* have been inadequate in comparison to those for *P. falciparum* (Price et al., 2009) and one of the main reasons behind the lag of *P. vivax* research is the inability of achieving a stable and long-term *in-vitro* culture for *P. vivax* leading to significantly restricted laboratory-based experimental studies. Advances in *in vitro* culture of *P. knowlesi* in human RBCs, have given critical support for more sophisticated laboratory investigations (Moon et al., 2013; Mohring et al., 2019), allowing some practical functional studies of *P. vivax* to be conducted*. Plasmodium* spp. other than *P. vivax* target almost all stages of RBCs, whereas *P. vivax* preferentially invades immature RBCs or reticulocytes (Kitchen, 1938), which normally account for 1-2% of the red blood cells in the peripheral blood circulation. Although advancement has been made in understanding the molecular basis underlying *P. vivax* reticulocyte preference for invasion (Gruszczyk et al., 2018a), still a powerful tool lacks (Krotoski et al., 1982; Mueller et al., 2009) which will help us in overcoming the difficulties in maintaining *P. vivax* in long-term cultures as it is relatively more difficult to repeatedly obtain and supplement reticulocyte-rich human blood to *P. vivax* cultures.

Another unique challenge with *P. vivax* is its ability to produce a dormant liver-stage forms or hypnozoites (Krotoski et al., 1982; Markus, 2011) which are responsible for multiple clinical relapses after a primary infection (Imwong et al., 2007). Prevention of *P. vivax* relapses is a must for *P. vivax* malaria to be eliminated. The distinction between relapse, recrudescence, and reinfection, and thus identifying early resistance, is a fundamental difficulty in therapeutic assessment. Chloroquine and the ACT companion medications are very slowly removed, so suppressive blood concentrations can last for weeks post medication (White, 2021).

Clinical intervention of *P. vivax* malaria requires clinical suspicion, an accurate blood test, and access to an efficient schizonticidal and hypnozoiticidal medication regimens. Although *P. vivax* is known to be still sensitive to chloroquine combined with primaquine, cases of chloroquine and sulfadoxine-pyrimethamine drug resistant *P. vivax* have also been reported from many areas of the globe including Australia, Ethiopia, Pakistan, Indonesia, Papua New Guinea, S. Korea and India (Rieckmann et al., 1989; Schunk et al., 2006; Price et al., 2009; Khatoon, 2010; Price, 2014). In addition, the main problem in managing a *P. vivax* infection is the management of frequent relapses for which both primaquine and its new counterpart, tafenoquine, have problems related to treatment adherence and safety with respect to G6PD deficiency. Thus, the unique clinical biology of *P. vivax* and restricted progress in the advancement of research tools (Su, 2019), create an obstacle in the way of growth of efficacious drugs and vaccines for vivax malaria. Adding to the above reasons, lack of financing, a paucity of resources and a high cost to create new vaccines contributes to the slow progress in case of development of a successful *P. vivax* vaccine.

## An Ideal *Plasmodium vivax* Malaria Vaccine

Regardless of decades of continuous efforts, only one vaccine (pre-erythrocytic vaccine RTS, S/ASO1 also known as Mosquirix) for *P. falciparum*, has been licensed for human use (RTS,S Clinical Trials Partnership, 2014; Laurens, 2020), but no vaccine for *P. vivax* is available yet. *Plasmodium* spp. exhibits a unique set of antigens at each stage of its life which makes it difficult for a researcher to identify the best vaccine candidate. The complex biology of *P. vivax*, its extensive antigenic diversity and its pathway of immune evasion make vaccine development against *P. vivax* malaria challenging. *P. vivax* is reported to exhibit greater genetic diversity in comparison to *P. falciparum* (Neafsey et al., 2012; Winter et al., 2015). While selecting a vaccine candidate for *P. vivax*, it is highly crucial to focus on those playing a role in invasion and those with a conserved epitope, which can be targeted by neutralizing the strain transcending antibodies. The discovery of broadly conserved inhibitory epitopes provides important new themes for the next generation of *P. vivax* malaria vaccines, as well as a foundation for rational structure-based vaccine design that will impart global strain-transcending protection (Chen et al., 2016). Multiple clinical isolates of *P. vivax* were used to investigate a panel of human monoclonal antibodies for their ability to inhibit *Pv*DBP from binding to the DARC, as well as their ability to impede red blood cell invasion and reticulocyte invasion. This led to the discovery of a widely neutralizing human monoclonal antibody that prevented *P. vivax* invasion in all tested strains (Rawlinson et al., 2019).

## Current Status of Candidate *P. vivax* Malaria Vaccines

The designing and distribution of a successful *P. vivax* vaccine tends to be a prime concern for speeding up malaria elimination in the Asia-Pacific and the Americas (Tanner et al., 2015). Only a few *P. vivax* vaccine candidates are close to or have reached different stages of clinical trials (Mueller et al., 2015; Draper et al., 2018). The delay in the development of a CSP-based vaccine for *P. falciparum*, RTS,S/AS01 (RTS,S) clearly indicates that much more work awaits for a comparable *P. vivax* vaccine. Although there is potential current research into *P. vivax* vaccine targets and immunisation tactics, the odds of a *P. vivax* vaccine becoming available in the near future are low. To date, human clinical trials have only been carried out for three *P. vivax* antigens namely, the *Pv*CSP-based pre-erythrocytic vaccine, the *Pv*DBP-based blood stage vaccine and the transmission-blocking candidate *Pv*s25 (Rainbow Tables, WHO). Several novel vaccine candidates are now being studied in a pre-clinical setting and there are excellent reviews discussing them (Galinski and Barnwell, 2008; Valencia et al., 2011).

The VMP001/AS01_B_ vaccine, which encompasses the N- and C- terminal regions of the CSP and a short repeat region comprising of repeat sequences from both the VK210 (type 1) and the VK247 (type 2) genotypes of *P. vivax* has been shown to clear the Phase I/IIa trial, increasing antibody and cell-mediated immune responses and subsequently resulting in a delay in the pre-patency period in 30 Duffy-positive vaccines (Bennett et al., 2016), but no sterile protection was achieved. However, a combination of *Pv*CSP and *Pv*TRAP provided sterile protection in mice using doses that individually conferred low or no protection (Atcheson et al., 2018). Phase II trials with another candidate, *Pv*RAS (*Plasmodium vivax* Radiation-Attenuated Sporozoites), showed immunogenic and sterile immunity in only 42% of the Duffy +ve (Fy+) subjects (Arevalo-Herrera et al., 2016). Transmission Blocking Vaccines targeting either a) pre-fertilization antigens expressed by gametocytes (*Pv*s48/45 and *Pv*s47) and gametes (*Pv*s230) (Sauerwein and Bousema, 2015; Tachibana et al., 2015) and b) post-fertilization antigens expressed by zygotes/ookinetes/oocysts (*Pv*s25 and *Pv*s28) (Hisaeda et al., 2000; Sauerwein and Bousema, 2015). To date, the *Pv*s25 protein present on the surface of ookinetes and oocysts (Tsuboi et al., 1998), is one of the best characterized Transmission Blocking Vaccine candidate Blagborough et al. (2016). Phase 1 trial using *Pv*s25 formulated with Montanide ISA 51 as an adjuvant has demonstrated significant antibody responses in volunteers, but trial was stopped due to frequent local reactogenicity such as erythema, induration, swelling, and tenderness at the site of injection (Wu et al., 2008). However, pre-clinical and clinical studies with P25 proteins shows that inclusion of a carrier protein could potentially boost its immunogenicity (Qian et al., 2007; Parzych et al., 2017; Radtke et al., 2017).

While evaluating a novel vaccine candidate antigen’s eligibility, it should be checked whether the gene that encodes it is required for parasite growth, as targeting a non-essential gene would appear to favour parasites that do not rely on the gene product and hence are immune to the vaccine. Although progress has been observed in identification and antigenic characterization of different *P. vivax* antigens, this review mainly focuses on the blood stage vaccine candidates and that too on *Pv*DBP, the only to-date blood stage vaccine candidate that has reached Phase 1 clinical trial (de Cassan et al., 2015; Bhardwaj et al., 2017; Payne et al., 2017; Singh et al., 2018). Antigens expressed on the merozoite surface are considered as blood stage vaccine targets. An effective vaccination against *P. vivax* blood stages would decrease symptoms and pathology associated with such repeated infections, and so potentially play a crucial role in controlling the species. In addition to provision of safety and efficacy, an ideal blood-stage vaccine candidate antigen should be capable of eliciting a strong immune response that inhibits *Plasmodium* from invading the target host cell. In comparison to 15 P*. falciparum*’s blood stage vaccine candidates that have been described in literature so far (Illingworth et al., 2019), only a few candidates have been studied in case of *P. vivax* (**Table 1**), including Duffy Binding Protein (*Pv*DBP), Merozoite Surface Protein 1 (MSP1), Apical Membrane Antigen 1 (*Pv*AMA1) and Reticulocyte Binding Protein (*Pv*RBP2b), a distant homologue of Reticulocyte Binding Protein Homologue 5 (*Pf*Rh5). Utilizing *P. knowlesi* as a screening model, research on a panel of *P. vivax* proteins (*Pv*MSP7.1, *Pv*MSP3.10, *Pv*12, *Pv*41, *Pv*GAMA, *Pv*CyRPA and *Pv*ARP) hypothesized to act in erythrocyte invasion, found an additional erythrocytic stage vaccine candidates (Ndegwa et al., 2021). Taking into account all of the benefits and drawbacks of any model system, it can be concluded that *P. knowlesi* might serve as an accessible and efficient model to screen for new candidates until a robust and long-term *P. vivax* culture is produced.

**Table 1:** *Plasmodium vivax* blood stage vaccine candidates.

	Description/delivery system	Development phase	Antigen	Reference
*Pv*DBPII/GLA-SE	Recombinant *Pv*DBPII with Glucopyranosyl Lipid Adjuvant-Stable Emulsion	Phase I b	*Pv*DBP	Bharadwaj et al., 2017; Singh et al., 2018
ChAd63-MVA *Pv*DBP RII	Prime boost, viral vectors (Chimpanzee Adenovirus 63/Modified Vaccinia Ankara)	Phase I a	*Pv*DBP	de Cassan et al., 2015; Payne et al., 2017
*Pv*DBPII-DEK^null^	Recombinant protein	Pre-clinical	*Pv*DBP	Ntumngia and Adams, 2012
*Pv*MSP1_19_	Recombinant protein-Montanide ISA720	Pre-clinical	*Pv*MSP1	Fonseca et al., 2016
ChAd63-*Pv*AMA1/MVA-*Pv*AMA1	Chimpanzee Adenovirus 63/Modified Vaccinia Ankara	Pre-clinical	*Pv*AMA1	Bouillet et al., 2011
*Pv*AMA1	Recombinant protein-adjuvant	Pre-clinical	*Pv*AMA1	Vicentin et al., 2014; Arévalo-Pinzón et al., 2017
*Pv*RBP2b	Recombinant protein	Pre-clinical	*Pv*RBP	Gruszczyk et al., 2018a, Gruszczyk et al., 2018b

To date, human trials in the erythrocytic stage have only been carried out for *Pv*DBP-based vaccine. *P. vivax* invasion of human RBCs is restricted to interaction of *Pv*DBP with human reticulocytes (via the Duffy Antigen Receptor for Chemokines, DARC) expressing the Iron Importer, Transferrin Receptor 1 (TfR1) or Cluster of Differentiation 71 (CD71) (Malleret et al., 2015). Till date, only two vaccines targeting the conserved cysteine-rich region II of *Pv*DBP have reached clinical trials, ChAd63/MVA *Pv*DBP RII (Payne et al., 2017) and *Pv*DBPII/GLA-SE (Bhardwaj et al., 2017; Singh et al., 2018).

## *Pv*DBP, an Essential Parasite Ligand for Human Reticulocyte Invasion

A number of distinct invasion pathways have been identified by *Plasmodium* spp. that exploit unique sets of human red blood cell (RBC) receptors for invasion. Two major protein families of *Plasmodium*, the **Erythrocyte-Binding-Like (EBL) family**, expressed from the *erythrocyte-binding-like* (*ebl*) genes (Fang et al., 1991) and the **Reticulocyte-Binding-Like (RBL) protein homologs (RBL or Rh)**, expressed from *reticulocyte binding protein* genes (Galinski et al., 1992) are responsible for parasite’s tight interactions with different stages of host’s RBCs. There exists a species-specific variation in the count of EBL proteins, *P. falciparum* having five members while *P. vivax* has only a single member (Adams et al., 1992; Adams et al., 2001). The EBL family further consists of the Duffy-Binding-Like (DBL-EBL) and Erythrocyte-Binding Protein sub-families (EBP) (Adams et al., 2001). The DBL-EBL proteins are characterized by presence of two cysteine-rich regions and a Duffy-binding domain in the N-terminal cysteine-rich region (Adams et al., 1992). On the other hand, the members of the RBL family solely target the reticulocytes as well as normocytes, producing parasite proteins which facilitate reticulocyte binding and/or invasion (Ntumngia et al., 2018). Reticulocyte-binding proteins (RBPs) were originally discovered in *P. vivax* (Galinski et al., 1992) and are the classic instances of reticulocyte binding-like/reticulocyte-binding homolog (RBL/RH) proteins, which have also been discovered in *P. cynomolgi* (Okenu et al., 2005) and *P. yoelii* (Ogun et al., 2011). In *P. vivax*, the RBL determine the reticulocyte restriction of this species. Out of the five *Pv*RBPs, only one (*Pv*RBP2b) is found to bind exclusively to reticulocytes (França et al., 2016).

## Duffy-Binding-Like Sub-family of Erythrocyte-Binding-Like Family

A huge macromolecular cascade of proteins is likely involved in host cell selection and invasion activities. However, just a few will be crucial participants in allowing the parasite to retain a significant red blood cell invasion capacity in the face of physiological and immunological changes in the host. This adds to the latency of the infection and, as a result, enhance the possibilities of transmission. These proteins are most likely parasite ligands involved in the erythrocyte surface binding events that contribute to effective invasion. Sequestered in the micronemes of merozoites, the DBL–EBPs are type-I membrane proteins which are supposed to be released during the invasion process (Adams et al., 1990). The first DBL-EBL was identified in *P. knowlesi* and was called Duffy-Binding Protein (*Pk*DBP) as it was shown to bind the Duffy Antigen Receptor for Chemokines (DARC) on RBCs (Chitnis and Miller, 1994). Subsequently, its orthologues in *P. vivax* and *P. falciparum* have also been identified (Haynes et al., 1988). Members of DBL-EBL family are characterized to have six extracellular regions (RI-RVI), subsequently followed by a type I trans-membrane domain, and a short cytoplasmic tail (Adams et al., 1992). Out of the six extracellular regions, the two hydrophobic cysteine-rich regions (N-terminal RII and C-terminal RVI) are functionally conserved in all erythrocyte binding proteins (EBLs) and separated by three low-homology regions (RIII-RV). The N-terminal cysteine-rich region (RII) carries the binding residues responsible for binding to the DARC (Chitnis and Miller, 1994), whereas the C-terminal cysteine-rich region (RVI) has no clear known function, although a high degree of amino acid conservation among the three *Plasmodium* species (*P. falciparum*, *P. vivax* and *P. knowlesi*) is observed which suggests that this domain might have some importance (Adams et al., 1992). *P. falciparum* and *P. knowlesi* exhibit a variety of proteins (*Pf*EBL-1, *Pf*EBA-140, *Pf*EBA-175, *Pf*EBA-181, *Pf*EBA-165, *Pk*DBPα and multiple DBP-like ligands) belonging to DBL-EBL family, creating alternative pathways of RBC invasion, whereas, *P. vivax* comprises of a single protein, *Pv*DBP (Adams et al., 1992) of the DBL-EBL family. *Pv*EBP, in addition to *Pv*DBP, is a new member to this family (Roesch et al., 2018).

**Figure 1:**
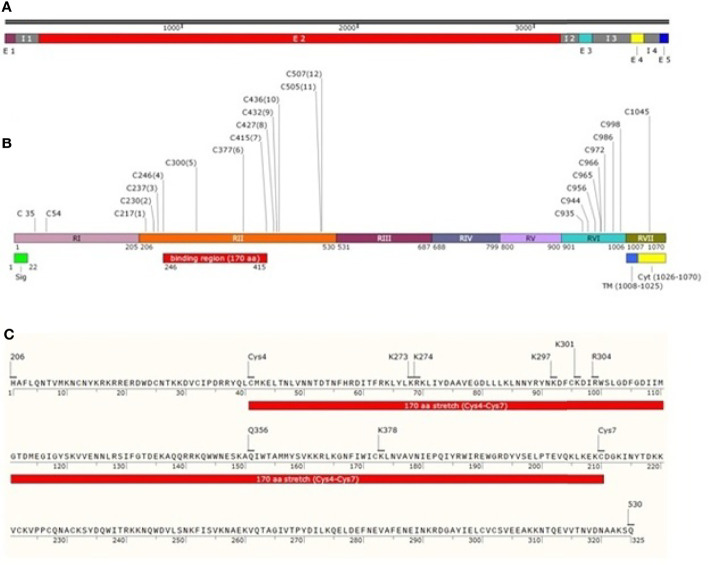
Schematic drawing of *Pv*DBP. **(A)** *Pvdbp* gene (3762 nucleotides) with 5 exons (1-57, 193-3151, 3257-3335, 3554-3627 and 3719-3762, respectively) and 4 introns (58-192, 3152-3256, 3336-3553 and 3628-3718, respectively). **(B)** *Pv*DBP consisting of 1070 amino acid residues. The shared boundaries for regions I-VII (205, 530, 687, 799, 900, 1006 and 1070 amino acids, respectively) (Adams et al., 1992; Okenu et al., 2005). Labelled in it are the positions corresponding to seven regions (RI-RVII) of *Pv*DBP, the cysteine-rich regions (RII and RVI), signal peptide shaded in green (1-22aa), transmembrane domain shaded in blue (1008-1025aa) and cytoplasmic domain shaded in yellow (1026-1070aa). *Pv*DBP consists of 23 cysteines (two in RI, twelve in RII, eight in RVI and one in RVII). The cysteines are labelled along with their amino acid position. The binding residues map to a 170 aa stretch which starts from Cys4 and ends at Cys7 (C246-C415) **(C)** *Pv*DBPII spans a length of 325 aa from 206-530aa (Adams et al., 1990). The *Pv*DBPII binding residues at sub domain 2: Site 1 (K297, K301, R304 and K378 (VanBuskirk et al., 2004b)) and Site 2 (K273, K274 and Q356 (Hans et al., 2005)) are responsible for reticulocyte binding with respect to 1070 aa residues of *Pv*DBP (NCBI protein id: XP_001608387.1).

*Plasmodium vivax* Duffy Binding Protein (*Pv*DBP) is a 140-kDa trans-membrane protein responsible for reticulocyte invasion of *P. vivax* and is dependent on the host’s Duffy Antigen Receptor for Chemokines (DARC) (Horuk et al., 1993). The *Pvdbp* gene (PlasmoDB Gene ID = *PV*X_110810) is present in chromosome 6 of *P. vivax* spanning a length of 3,762 nucleotides Carlton et al. (2008) (**Figure 1A**) and comprising of five exons and four introns (Fang et al., 1991). Exon 1 (57 nucleotides) of *Pvdbp* encodes a signal sequence, exon 2 (2,959 nucleotides) encodes 986 amino acids and covers the six extracellular regions, RI-RVI of the translated protein, exon 3 spans 79 nucleotides and comprises a trans-membrane domain (18 amino acids), exons 4 and 5 spanning 74 and 44 nucleotides, respectively. Exons 4 and 5 and a portion of exon 3 translates into a cytoplasmic tail (45 amino acids) (Adams et al., 1990; Adams et al., 1992) (**Figure 1B**). The N-terminal cysteine rich region (RII) comprises of DBL domains (Chitnis and Miller, 1994)which contain binding residues responsible for formation of tight junction between *Pv*DBP and DARC. The C-terminal cysteine-rich region (RVI), is separated from RII by three hydrophilic regions III, IV and V and is followed by the trans-membrane domain.

The N-terminal cysteine rich region (RII) of *Pv*DBP starts and ends at H206 and Q530, respectively (VanBuskirk et al., 2004b) (**Figure 1C**). It has been found that RII spans 325 aa residues, and not 330 aa, as it was thought previously. This 325 aa region (RII) comprises of 12 conserved cysteine residues (C217, C230, C237, C246, C300, C377, C415, C427, C432, C436, C505, and C507) (Fang et al., 1991; Adams et al., 1992). The cysteines are reported to contribute to DBL’s structural integrity (Singh et al., 2003; Singh et al., 2006), and so the parasite may not afford changes in these residues. The region deepest within the DBL domain, i.e. between cysteines 4 and 8, have been marked as the portion bearing the prime components for receptor recognition (Tsuboi et al., 1994; Ranjan and Chitnis, 1999; Xainli et al., 2000). The minimal binding region of *Pv*DBPII to the human DARC is localized between cysteines 4 and 7 (Ranjan and Chitnis, 1999; Batchelor et al., 2011). Residues between cysteines 7 and 8 are supposed to be surface-exposed and are not significantly involved in receptor binding (Ranjan and Chitnis, 1999; VanBuskirk et al., 2004b).

*Pk*α/*Pv*-DBL is a compact helical, monomeric module spread over three distinct subdomains (SD1, SD2 and SD3). *Pk*α/*Pv*-DBL consists of twelve cysteine residues which are stabilized by intra-domain disulfide bonds mostly conserved amongst the DBL family of EBPs (Singh et al., 2006). The indispensable and invariant residues required for DARC recognition (**Figure 1C**) were mapped within a region on SD2 (Singh et al., 2003; Hans et al., 2005; Singh et al., 2006; Yogavel et al., 2018), which lies between Cys4 (C246) - Cys7 (C415). Cys1 (C217) - Cys3 (C237) and Cys8 (C427) - Cys12 (C507) which flank SD2 (Cys4-Cys7) might play a structural role in the intact DBL domain (Ranjan and Chitnis, 1999; Singh et al., 2003; Singh et al., 2006) (**Table 2**). SD1 is not required for DBL-DARC interaction (Singh et al., 2003) whereas the functional significance of SD3 is still in question.

**Table 2:** *Pv*DBPII separated into 3 sub-domains.

	Amino acid residues	No. of Intra sub-domain sulfides
Sub-domain 1	N211-L253	2 (C217–C246 and C230–C237)
Sub-domain 2	Y271-E386	1 (C300–C377)
Sub-domain 3	P387–S508	3 (C415–C432, C427–C507 and C436–C505)

Although region II plays a significant role in receptor recognition, this region with respect to the rest of *Pvdbp* gene is hyper-variable with a high ratio of non-synonymous to synonymous mutations (Tsuboi et al., 1994; Xainli et al., 2000; Cole-Tobian and King, 2003), which might be one of the factors which help the parasite to escape host immunity (Tsuboi et al., 1994; Xainli et al., 2000). Exploration of *PvdbpII* genetic variation among *P. vivax* endemic regions showed that *Pv*DBPII is highly polymorphic, however, no changes in the cysteine residues have been reported so far (Tsuboi et al., 1994; Ampudia et al., 1996; Xainli et al., 2000; Kho et al., 2001; Cole-Tobian and King, 2003; Sousa et al., 2006; Gosi et al., 2008; Babaeekhou et al., 2009; Batchelor et al., 2011; Premaratne et al., 2011; Chenet et al., 2012; Ju et al., 2012; Ju et al., 2013.

The polymorphic residues adjacent to the binding site are reported to escape the binding inhibitory antibodies thus keeping the binding site of the protein undisturbed. Site-directed mutagenesis of *Pv*DBPII identified several residues which are vital for receptor recognition (VanBuskirk et al., 2004b). The conserved residues present in the binding region of *Pv*DBP were found to be responsible for ligand receptor interaction. The variant residues are reported to flank the functionally important residues. So, changes occurring in the conserved amino acid residues (which are not exposed on the surface, as a result are not detected by hosts immunity) might be accountable for loss of binding activity. The reported polymorphic residues were not found to affect reticulocyte binding as they are found to be mapped in the face opposite to the residues critical for binding to DARC (Chitnis and Sharma, 2008).

Batchelor *et al.* elucidated the crystal structure of *Pv*DBPII (PDB: 3RRC), which indicates a model for receptor recognition through *Pv*DBP dimerization, facilitating the development of a complex composed of two *Pv*DBP and two DARC molecules, which might pave way towards invasion (Batchelor et al., 2011). The critical binding residues required for reticulocyte binding were found to be structurally and functionally conserved, and are also targets of immune response (Batchelor et al., 2011). Protective antibodies targeting the critical binding regions in *Pv*DBPII were found to disturb dimerization and/or inhibit receptor binding. A step-wise binding model has also been proposed which involves receptor-induced *Pv*DBPII dimerization facilitating the formation of a heterotrimer that eventually employs a second DARC molecule to form a heterotetramer (PDB: 4NUU and 4NUV) Batchelor et al. (2014). Although these structural and biophysical studies provide deep insight into *Pv*DBPII-DARC engagement, further studies are required to assess these models as this region is prone to polymorphisms (Mittal et al., 2020) and as a result, the inherent variability in *Pv*DBL might render the *Pv*DBP-based vaccines inefficacious. Further, Yogavel et al. reported the existence of two binding sites in *Pv*DBPII, a) Site 1 which includes residues K266, K270, R273 and K347 and, b) Site 2 including residues K242, R243 and H325 (from PDB: 3RRC, 4NUU and 4NUV) (Yogavel et al., 2018). The DARC peptide, by means of its sulfated Tyr41 and phosphated Tyr30, engages at sites 1 and 2, respectively on *Pv*/*Pk*-DBLs. This is a testable model depicting DARC’s engagement with *Pv*/*Pk*-DBP and needs to be experimentally assessed for further confirmation.

Keeping allelic variation in mind, a successful and efficacious DBPII-based vaccine should aim at conserved epitopes which are supposed to be the prospective targets of strain-transcending neutralizing immunity. Naturally acquired binding-inhibitory antibodies to *Pv*DBPII are associated with clinical immunity of the subject to *P. vivax* malaria and thus potently neutralize the *P. vivax* invasion mechanism (Grimberg et al., 2007; Chootong et al., 2010; Nicolete et al., 2016)

**Figure 2:**
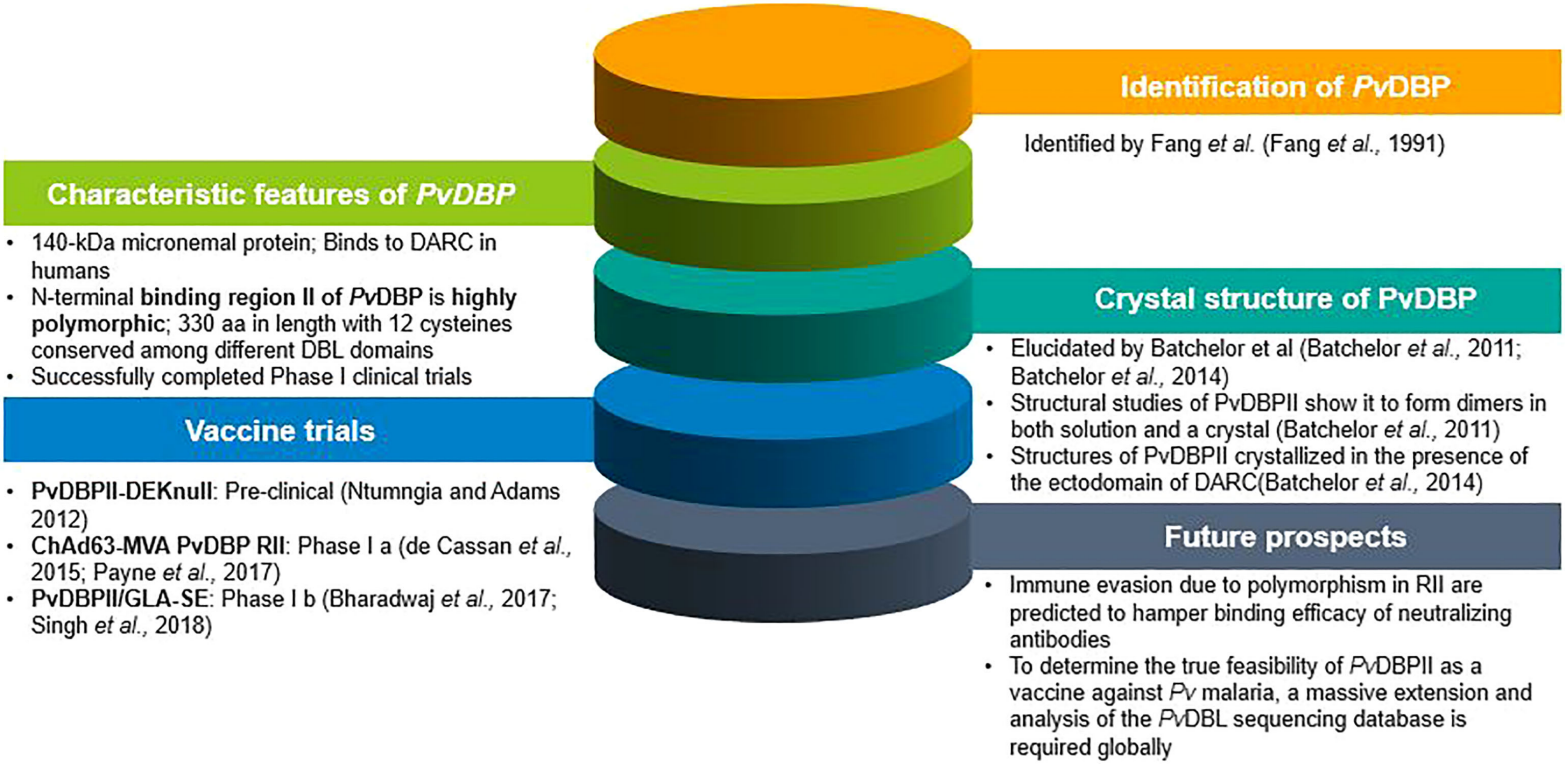
Pictorial representation of landmarks in PvDBP research.

For the first time, studies using ELISA and flow cytometry confirmed that both rabbit and human antibodies inhibited recombinant *Pv*DBPII-DARC interactions and were found to reduce invasion efficiency of wild *P. vivax* by up to 64%, while a reduced *P. vivax* invasion by up to 54% was observed in a combined *Pv*DBPII antisera from people exposed to *P. vivax* (Grimberg et al., 2007). Polymorphisms in *Pv*DBPII and the presence of multiple strains in endemic regions present unique challenges in the path of vaccine design (VanBuskirk et al., 2004a; Cole-Tobian et al., 2009; Ntumngia et al., 2012). In spite of the variations that exists in *Pv*DBPII, broadly conserved epitopes of three inhibitory murine monoclonal antibodies have been recognized in *Pv*DBPII (subdomain 3) (Chen et al., 2016) which were not found to lie in close vicinity to the dimer interface as well as the DARC-binding site (Chen et al., 2016). Clinical trials in humans using *Pv*DBPII produced antibodies that block *in vitro* binding of different allelic variants of *Pv*DBPII to the DARC for more than 100 days following three immunization doses (Payne et al., 2017; Singh et al., 2018). Both the vaccine candidates of *Pv*DBP which are in clinical trial (*Pv*DBPII/GLA-SE, ChAd63-MVA *Pv*DBP RII) were found to give rise to strain-transcending antibodies. By means of human mAbs produced in the course of vaccination or through natural *P. vivax* exposure, a broadly neutralizing human mAb have been identified which inhibited the invasion of all tested strains of *P. vivax*, thus indicating the molecular basis for inhibition. that will thus aid in the design of successful and efficient DBP-based vaccine for *P. vivax* malaria (Rawlinson et al., 2019; Urusova et al., 2019). The various landmarks achieved in PvDBP research are shown in **Figure 2**.

## Duffy Negativity and Other Challenges

The Duffy antigens act as receptors for a wide range of chemokines and are therefore called as Duffy Antigen Receptor for Chemokines (DARC). The same Duffy antigens also serve as receptors for *P. knowlesi*, *P. vivax and P. cynomolgi* (Kosaisavee et al., 2017). Absence of DARC on the reticulocyte surface is thought to confer protection against blood stage infections caused by *P. vivax* in Africa (Sanger et al., 1955; Miller et al., 1976). However, Duffy-negative individuals infected with *P. vivax* have been reported in sub-Saharan Africa (Ryan et al., 2006; Gosi et al., 2008; Ménard et al., 2010; Woldearegai et al., 2013; Djeunang Dongho et al., 2021), which points to the fact that there might be an alternative route of invading human reticulocytes lacking DARC. A recent study conducted in 952 individuals observed the absence of *P. vivax* infections in Ghana where a high frequency of the Duffy-negative genotype was reported (Brown et al., 2021). Human *P. vivax* strains have been reported in Madagascar and parts of Africa, which might be due to the re-establishment of this parasite (Culleton and Carter, 2012). However, it is still to be confirmed whether these cases have emerged due to the introduction of a new *P. vivax* strain which may use an alternative pathway which is independent of DARC. Of late, a novel *P. vivax* Erythrocyte Binding Protein (*Pv*EBP, also known as DBP2) was reported which may enable interactions with other membrane proteins on erythrocytes (Hester et al., 2013). *Pvebp* gene belongs to the DBL-EBP family, which harbours all the key features of EBPs, suggesting its ability to bind to human erythrocytes and facilitate RBC invasion (106). This gene was found to be expressed in the blood-stage of *P. vivax* and was found in all *P. vivax* strains examined, comprising an extensive geographical span (Hester et al., 2013). Both *Pv*DBP and *Pv*EBP were found to be antigenically distinct. The discovery of *Pv*EBP promptly highlights an alternative invasion pathway and could perhaps illuminate the initial step towards decoding the principle underlying *P. vivax* infection of the Duffy-negatives. Further investigations on *Pv*EBP revealed its preferential binding to young (CD71^high^) Duffy positive reticulocytes and minimal binding capacity for Duffy-negative reticulocytes (Ntumngia et al., 2016). This study proposes that *Pv*EBP might not serve as a ligand for Duffy-negative reticulocytes, but may act as an alternative pathway for invading Duffy-positive population. Whole genome sequencing studies show that *Pvdbp* gene is duplicated (at a higher rate) in Madagascar population, where both Duffy-negative and Duffy-positive individuals co-exist (Menard et al., 2013), and was supposed to be associated with infection in Duffy negatives possibly in response to constraints imposed by Duffy negativity in some human populations. In addition to this another study confirmed the presence of multiple copies of *Pvdbp* gene (3 and 8 copies) in two Duffy-negative Ethiopian isolates (Gunalan et al., 2016; Roesch et al., 2018). But this observation was contradicted by emergence of widespread *Pvdbp* gene duplication in malaria endemic areas of South-east Asia comprising Duffy-positive population (Hostetler et al. (2016). Although an excess of nonsynonymous mutations and no synonymous mutations was observed in *Pvebp* in comparison to *Pvdbp*, but in terms of allelic diversity, *Pvebp* was found to be less diverse than *Pvdbp* in Madagascar (both Duffy-negative and Duffy-positive) and Cambodian population (Duffy-positive). The absence of synonymous mutation in this case clearly marks that the *Pvebp* gene is under strong positive selection and validates the importance of this protein in reticulocyte invasion as well as Duffy-independent invasion pathways used by ***Plasmodium vivax*** (Roesch et al., 2018).

## Conclusion

*P. vivax* has evolved with a variety of mechanisms to overcome immune defense at every step of communication with its host species. Developing an effective vaccine that provides protection and prevents transmission is highly essential in eliminating *P. vivax* malaria. The Region II of *Pv*DBP, the only blood-stage vaccine candidate, spans 325 aa residues, and not 330 aa, as was previously reported. *Pv*DBPII is the lone vaccine candidate that has entered clinical trial Phase 1b and is crucially involved in *P. vivax* merozoite invasion of human reticulocytes.

In spite of the fact that *Pv*DBP is crucial for blood-stage infection, its exercise for vaccine development constitutes of major obstacles as cited below.

(i) Polymorphisms in *Pv*DBP seem to be critical for the evasion of host immune response. A brief exposure of *Pv*DBP to the host’s immune system, due to its micronemal location and the rapid kinetics of parasite invasion, allows a short-term exposure of *Pv*DBP to the host immune system. As a result of this phenomenon, the parasite might gain an advantage of escaping the host’s immunity. Immune selection being a major driving force for allelic variation, as even a single amino acid substitution can change the antigenic nature of a pathogen. Also the evolutionary arms race between the parasite and human indicates that the parasite genome is evolving at a higher rate than the latter. The amount of polymorphic data available globally is insufficient for declaring *Pv*DBP as a long-lasting and effective vaccine candidate. Therefore, a global (covering the *P. vivax* endemic regions) rigorous survey in terms of allelic diversity of *Pv*DBL domain is required. All these points lead to the fact that we are in requirement of a candidate antigen that is less polymorphic.
(ii) The surfacing of *P. vivax* infection in Duffy negative population is an alarming condition.
(iii) Emergence of newly reported *P. vivax* ligands targeting RBCs questions the viability of *Pv*DBPII as the lone vaccine candidate and (iv) presence of more than single copy (1-4 reported) of *Pvdbp* might create an obstacle in the way of attaining an efficacious vaccine.
(iv) A strain transcending *Pv*DBPII-based vaccine demands a globally conserved epitope. Mittal *et al.*, in their global Single Amino Acid Polymorphism (SAAP) data analysis reported that from the four *Pv*DBL–mAb complex structures, 2 out of the 4 purported neutralizing mAbs do not bind near the supposed dimer interface (Mittal et al., 2020). Such discoveries points towards the fact that a vaccine against *P. vivax* could have more impact if above challenges of *Pv*DBPII (as the lone candidate) were considered.
(v) Considering *Pv*DBPII as a potential vaccine target, its immunodominant variant epitopes deflect immune responses, compromising the vaccine efficacy in triggering high titer neutralising antibodies against conserved strain-transcending functional epitopes (Chootong et al., 2010; Ntumngia and Adams, 2012).

Contributing to the formulation of preventative and/or therapeutic approaches which will assist in minimising the effects of malaria, there is a requirement of deciphering and combining functional and structural investigations (Patarroyo et al., 2020).While *Pv*DBP may still be required for the invasion of Duffy negative erythrocytes (Gunalan et al., 2018; Lo et al., 2019), the only focus on *Pv*DBP as a vaccine candidate certainly needs to be reconsidered, and alternative targets explored as potential substitutes for *Pv*DBP or in conjunction with it. A vaccine targeting only a single-stage parasite antigen faces challenges in retaining similar antibody responses due to the genomic changes in parasite ligands which in turn might improve the fitness of *P. vivax* isolates. Most of the *P. vivax* vaccines in pipeline target individual stages and are based on single antigens. Combination allele vaccines in case of *PvdbpII* achieve greater specificity by targeting a majority of antibody to common epitopes among the constituent alleles that form the vaccine (De et al., 2021; Ntumngia et al., 2013). This suggested that a vaccine with multiple DBPII variant alleles is necessary for broader coverage. For finding new interaction hotspots to which malaria elimination approaches can be directed, a profound analysis is needed to correlate structural, functional (adhesion, invasion, and inhibition), and polymorphism data (Patarroyo et al., 2020). Moreover, a blood stage vaccine has to face a huge number of merozoites in comparison to a few as in case of pre-erythrocytic and transmission blocking stage. So, it is of high importance to combine antigens including multi-stages of parasite life cycle to attain the purpose of developing an effective vaccine for *P. vivax* malaria.

## Author Contributions

AS conceptualized the study, coordinated, drafted and critically revised the manuscript. SK aided in conception, literature search and drafting the whole manuscript. All authors gave final approval for publication and agree to be held accountable for the work performed therein.

## Conflict of Interest

The authors declare that the research was conducted in the absence of any commercial or financial relationships that could be construed as a potential conflict of interest.

## Publisher’s Note

All claims expressed in this article are solely those of the authors and do not necessarily represent those of their affiliated organizations, or those of the publisher, the editors and the reviewers. Any product that may be evaluated in this article, or claim that may be made by its manufacturer, is not guaranteed or endorsed by the publisher.

## References

[B1] Adams J. H. Hudson D. E. Torii M. Ward G. E. Wellems T. E. Aikawa M.. (1990). The Duffy Receptor Family of Plasmodium Knowlesi is Located Within the Micronemes of Invasive Malaria Merozoites. Cell 63 (1), 141–153. doi: 10.1016/0092-8674(90)90295-p 2170017

[B2] Adams J. H. Sim B. K. Dolan S. A. Fang X. Kaslow D. C. Miller L. H.. (1992). A Family of Erythrocyte Binding Proteins of Malaria Parasites. Proc. Natl. Acad. Sci. U.S.A. 89 (15), 7085–7089. doi: 10.1073/pnas.89.15.7085 1496004 PMC49650

[B3] Adams J. H. Blair P. L. Kaneko O. Peterson D. S.. (2001). An Expanding Ebl Family of Plasmodium Falciparum. Trends Parasitol. 17 (6), 297–299. doi: 10.1016/s1471-4922(01)01948-1 11378038

[B4] Ampudia E. Patarroyo M. A. Patarroyo M. E. Murillo L. A. (1996). Genetic Polymorphism of the Duffy Receptor Binding Domain of Plasmodium Vivax in Colombian Wild Isolates. Mol. Biochem. Parasitol. 78 (1-2), 269–272. doi: 10.1016/s0166-6851(96)02611-4 8813697

[B5] Arévalo-Herrera M. Vásquez-Jiménez J. M. Lopez-Perez M. Vallejo A. F. Amado-Garavito A. B. Céspedes N.. (2016). Protective Efficacy of Plasmodium Vivax Radiation-Attenuated Sporozoites in Colombian Volunteers: A Randomized Controlled Trial. PLoS Negl. Trop. Dis. 10 (10), e0005070. doi: 10.1371/journal.pntd.0005070 27760143 PMC5070852

[B6] Arévalo-Pinzón G. Bermúdez M. Hernández D. Curtidor H. Patarroyo M. A. (2017). Plasmodium Vivax Ligand-Receptor Interaction: PvAMA-1 Domain I Contains the Minimal Regions for Specific Interaction With CD71+ Reticulocytes. Sci. Rep. 7 (1), 9616. doi: 10.1038/s41598-017-10025-6 28855657 PMC5577344

[B7] Atcheson E. Bauza K. Salman A. M. Alves E. Blight J. Viveros-Sandoval M. E.. (2018). Tailoring a Plasmodium Vivax Vaccine To Enhance Efficacy Through a Combination of a CSP Virus-Like Particle and TRAP Viral Vectors. Infect. Immun. 86 (9), e00114–e00118. doi: 10.1128/IAI.00114-18 29986894 PMC6105880

[B8] Babaeekhou L. Zakeri S. Djadid N. D.. (2009). Genetic Mapping of the Duffy Binding Protein (DBP) Ligand Domain of Plasmodium Vivax From Unstable Malaria Region in the Middle East. Am. J. Trop. Med. Hyg. 80 (1), 112–118. doi: 10.4269/ajtmh.08-0241err 19141848

[B9] Baird J. K.. (2007). Neglect of Plasmodium Vivax Malaria. Trends Parasitol. 23 (11), 533–539. doi: 10.1016/j.pt.2007.08.011 17933585

[B10] Baird J. K.. (2013a). Malaria Caused by Plasmodium Vivax: Recurrent, Difficult to Treat, Disabling, and Threatening to Life–the Infectious Bite Preempts These Hazards. Pathog. Glob. Health 107 (8), 475–479. doi: 10.1179/2047772413Z.000000000179 24428831 PMC4073528

[B11] Baird J. K.. (2013b). Evidence and Implications of Mortality Associated With Acute Plasmodium Vivax Malaria. Clin. Microbiol. Rev. 26 (1), 36–57. doi: 10.1128/CMR.00074-12 23297258 PMC3553673

[B12] Batchelor J. D. Zahm J. A. Tolia N. H.. (2011). Dimerization of Plasmodium Vivax DBP is Induced Upon Receptor Binding and Drives Recognition of DARC. Nat. Struct. Mol. Biol. 18 (8), 908–914. doi: 10.1038/nsmb.2088 21743458 PMC3150435

[B13] Batchelor J. D. Malpede B. M. Omattage N. S. DeKoster G. T. Henzler-Wildman K. A. Tolia N. H.. (2014). Red Blood Cell Invasion by Plasmodium Vivax: Structural Basis for DBP Engagement of DARC. PLoS Pathog. 10 (1), e1003869. doi: 10.1371/journal.ppat.1003869 24415938 PMC3887093

[B14] Battle K. E. Lucas T. Nguyen M. Howes R. E. Nandi A. K. Twohig K. A.. (2019). Mapping the Global Endemicity and Clinical Burden of Plasmodium Vivax 2000-17: A Spatial and Temporal Modelling Study. Lancet (London England) 394 (10195), 332–343. doi: 10.1016/S0140-6736(19)31096-7 PMC6675736 31229233

[B15] Bennett J. W. Yadava A. Tosh D. Sattabongkot J. Komisar J. Ware L. A.. (2016). Phase 1/2a Trial of Plasmodium Vivax Malaria Vaccine Candidate VMP001/AS01B in Malaria-Naive Adults: Safety, Immunogenicity, and Efficacy. PLoS Negl. Trop. Dis. 10 (2), e0004423. doi: 10.1371/journal.pntd.0004423 26919472 PMC4769081

[B16] Bhardwaj R. Shakri A. R. Hans D. Gupta P. Fernandez-Becerra C. Del Portillo H. A.. (2017). Production of Recombinant PvDBPII, Receptor Binding Domain of Plasmodium Vivax Duffy Binding Protein, and Evaluation of Immunogenicity to Identify an Adjuvant Formulation for Vaccine Development. Protein Expr. Purif. 136, 52–57. doi: 10.1016/j.pep.2015.06.011 26578115

[B17] Blagborough A. M. Musiychuk K. Bi H. Jones R. M. Chichester J. A. Streatfield S.. (2016). Transmission Blocking Potency and Immunogenicity of a Plant-Produced Pvs25-Based Subunit Vaccine Against Plasmodium Vivax. Vaccine 34 (28), 3252–3259. doi: 10.1016/j.vaccine.2016.05.007 27177945 PMC4915602

[B18] Bouillet L. É. Dias M. O. Dorigo N. A. Moura A. D. Russell B. Nosten F.. (2011). Long-Term Humoral and Cellular Immune Responses Elicited by a Heterologous Plasmodium Vivax Apical Membrane Antigen 1 Protein Prime/Adenovirus Boost Immunization Protocol. Infect. Immun. 79 (9), 3642–3652. doi: 10.1128/IAI.05048-11 21730090 PMC3165491

[B19] Brasil P. Zalis M. G. de Pina-Costa A. Siqueira A. M. Júnior C. B. Silva S.. (2017). Outbreak of Human Malaria Caused by Plasmodium Simium in the Atlantic Forest in Rio De Janeiro: A Molecular Epidemiological Investigation. Lancet Glob. Health Engl. 5 (10), e1038–e1046. doi: 10.1016/S2214-109X(17)30333-9 28867401

[B20] Brown C. A. Pappoe-Ashong P. J. Duah N. Ghansah A. Asmah H. Afari E.. (2021). High Frequency of the Duffy-Negative Genotype and Absence of Plasmodium Vivax Infections in Ghana. Malar. J. 20, 99. doi: 10.1186/s12936-021-03618-0 33596926 PMC7888148

[B21] Carlton J. M. Adams J. H. Silva J. C. Bidwell S. L. Lorenzi H. Caler E.. (2008). Comparative Genomics of the Neglected Human Malaria Parasite Plasmodium Vivax. Nature 455 (7214), 757–763. doi: 10.1038/nature07327 18843361 PMC2651158

[B22] Chen E. Salinas N. D. Huang Y. Ntumngia F. Plasencia M. D. Gross M. L.. (2016). Broadly Neutralizing Epitopes in the Plasmodium Vivax Vaccine Candidate Duffy Binding Protein. Proc. Natl. Acad. Sci. U.S.A. 113 (22), 6277–6282. doi: 10.1073/pnas.1600488113 27194724 PMC4896725

[B23] Chenet S. M. Tapia L. L. Escalante A. A. Durand S. Lucas C. Bacon D. J. (2012). Genetic Diversity and Population Structure of Genes Encoding Vaccine Candidate Antigens of Plasmodium Vivax. Malaria J. 11, 68. doi: 10.1186/1475-2875-11-68 PMC3330009 22417572

[B24] Chitnis C. E. Miller L. H.. (1994). Identification of the Erythrocyte Binding Domains of Plasmodium Vivax and Plasmodium Knowlesi Proteins Involved in Erythrocyte Invasion. J. Exp. Med. 180 (2), 497–506. doi: 10.1084/jem.180.2.497 8046329 PMC2191600

[B25] Chitnis C. E. Sharma A.. (2008). Targeting the Plasmodium Vivax Duffy-Binding Protein. Trends Parasitol. 24 (1), 29–34. doi: 10.1016/j.pt.2007.10.004 18023618

[B26] Chootong P. Ntumngia F. B. VanBuskirk K. M. Xainli J. Cole-Tobian J. L. Campbell C. O.. (2010). Mapping Epitopes of the Plasmodium Vivax Duffy Binding Protein With Naturally Acquired Inhibitory Antibodies. Infect. Immun. 78 (3), 1089–1095. doi: 10.1128/IAI.01036-09 20008533 PMC2825952

[B27] Chu C. S. White N. J.. (2021). The Prevention and Treatment of Plasmodium Vivax Malaria. PLoS Med. 18 (4), e1003561. doi: 10.1371/journal.pmed.1003561 33891587 PMC8064578

[B28] Cole-Tobian J. L. Michon P. Biasor M. Richards J. S. Beeson J. G. Mueller I.. (2009). Strain-Specific Duffy Binding Protein Antibodies Correlate With Protection Against Infection With Homologous Compared to Heterologous Plasmodium Vivax Strains in Papua New Guinean Children. Infect. Immun. 77 (9), 4009–4017. doi: 10.1128/IAI.00158-09 19564376 PMC2737995

[B29] Cole-Tobian J. King C. L.. (2003). Diversity and Natural Selection in Plasmodium Vivax Duffy Binding Protein Gene. Mol. Biochem. Parasitol. 127 (2), 121–132. doi: 10.1016/s0166-6851(02)00327-4 12672521

[B30] Conway D. J.. (2007). Molecular Epidemiology of Malaria. Clin. Microbiol. Rev. 20 (1), 188–204. doi: 10.1128/CMR.00021-06 17223628 PMC1797638

[B31] Culleton R. Carter R.. (2012). African Plasmodium Vivax: Distribution and Origins. Int. J. Parasitol. 42 (12), 1091–1097. doi: 10.1016/j.ijpara.2012.08.005 23017235

[B32] Deane L. M.. (1992). Simian Malaria in Brazil. Mem. Inst. Oswaldo Cruz Brazil 87 Suppl 3, 1–20. doi: 10.1590/s0074-02761992000700001 1343676

[B33] De S. L. Ntumngia F. B. Nicholas J. Adams J. H.. (2021). Progress Towards the Development of a P. Vivax Vaccine. Expert Rev Vaccines 20 (2), 97–112. doi: 10.1080/14760584.2021.1880898 33481638 PMC7994195

[B34] de Cassan S. C. Shakri A. R. Llewellyn D. Elias S. C. Cho J. S. Goodman A. L.. (2015). Preclinical Assessment of Viral Vectored and Protein Vaccines Targeting the Duffy-Binding Protein Region II of Plasmodium Vivax. Front. Immunol. 6. doi: 10.3389/fimmu.2015.00348 PMC4495344 26217340

[B35] Djeunang Dongho G. B. Gunalan K. L'Episcopia M. Paganotti G. M. Menegon M. Efeutmecheh Sangong R.. (2021). Plasmodium Vivax Infections Detected in a Large Number of Febrile Duffy-Negative Africans in Dschang, Cameroon. Am. J. Trop. Med. Hyg. 104 (3), 987–992. doi: 10.4269/ajtmh.20-1255 33534776 PMC7941824

[B36] Draper S. J. Sack B. K. King C. R. Nielsen C. M. Rayner J. C. Higgins M. K.. (2018). Malaria Vaccines: Recent Advances and New Horizons. Cell Host Microbe 24 (1), 43–56. doi: 10.1016/j.chom.2018.06.008 30001524 PMC6054918

[B37] Fang X. D. Kaslow D. C. Adams J. H. Miller L. H. (1991). Cloning of the Plasmodium Vivax Duffy Receptor. Mol. Biochem. Parasitol. 44 (1), 125–132. doi: 10.1016/0166-6851(91)90228-x 1849231

[B38] Fonseca J. A. Cabrera-Mora M. Singh B. Oliveira-Ferreira J. da Costa Lima-Junior J. Calvo-Calle J. M.. (2016). A Chimeric Protein-Based Malaria Vaccine Candidate Induces Robust T Cell Responses Against Plasmodium Vivax MSP119. Sci. Rep. 6, 34527. doi: 10.1038/srep34527 27708348 PMC5052570

[B39] França C. T. He W. Q. Gruszczyk J. Lim N. T. Lin E. Kiniboro B.. (2016). Plasmodium Vivax Reticulocyte Binding Proteins Are Key Targets of Naturally Acquired Immunity in Young Papua New Guinean Children. PLoS Negl. Trop. Dis. 10 (9), e0005014. doi: 10.1371/journal.pntd.0005014 27677183 PMC5038947

[B40] Galinski M. R. Barnwell J. W. (2008). Plasmodium Vivax: Who Cares? Malar. J. 7, S9. doi: 10.1186/1475-2875-7-S1-S9 19091043 PMC2604873

[B41] Galinski M. R. Medina C. C. Ingravallo P. Barnwell J. W.. (1992). A Reticulocyte-Binding Protein Complex of Plasmodium Vivax Merozoites. Cell 69 (7), 1213–1226. doi: 10.1016/0092-8674(92)90642-p 1617731

[B42] Gosi P. Khusmith S. Khalambaheti T. Lanar D. E. Schaecher K. E. Fukuda M. M.. (2008). Polymorphism Patterns in Duffy-Binding Protein Among Thai Plasmodium Vivax Isolates. Malaria J. 7, 112. doi: 10.1186/1475-2875-7-112 PMC2443374 18582360

[B43] Grimberg B. T. Udomsangpetch R. Xainli J. McHenry A. Panichakul T. Sattabongkot J.. (2007). Plasmodium Vivax Invasion of Human Erythrocytes Inhibited by Antibodies Directed Against the Duffy Binding Protein. PLoS Med. 4 (12), e337. doi: 10.1371/journal.pmed.0040337 18092885 PMC2140086

[B44] Gruszczyk J. Kanjee U. Chan L. J. Menant S. Malleret B. Lim N.. (2018a). Transferrin Receptor 1 Is a Reticulocyte-Specific Receptor for Plasmodium Vivax. Science (New York N.Y.) 359 (6371), 48–55. doi: 10.1126/science.aan1078 PMC5788258 29302006

[B45] Gruszczyk J. Huang R. K. Chan L. J. Menant S. Hong C. Murphy J. M.. (2018b). Cryo-EM Structure of an Essential Plasmodium Vivax Invasion Complex. Nature 559 (7712), 135–139. doi: 10.1038/s41586-018-0249-1 29950717

[B46] Gunalan K. Lo E. Hostetler J. B. Yewhalaw D. Mu J. Neafsey D. E.. (2016). Role of Plasmodium Vivax Duffy-Binding Protein 1 in Invasion of Duffy-Null Africans. Proc. Natl. Acad. Sci. U.S.A. 113 (22), 6271–6276. doi: 10.1073/pnas.1606113113 27190089 PMC4896682

[B47] Gunalan K. Niangaly A. Thera M. A. Doumbo O. K. Miller L.H.. (2018). Plasmodium Vivax Infections of Duffy-Negative Erythrocytes: Historically Undetected or a Recent Adaptation? Trends Parasitol. 34 (5), 420–429. doi: 10.1016/j.pt.2018.02.006 29530446 PMC6347384

[B48] Haldane J. B.. (2004). The Rate of Spontaneous Mutation of a Human Gene. 1935. J. Genet. 83 (3), 235–244. doi: 10.1007/BF02717892 15689625

[B49] Hans D. Pattnaik P. Bhattacharyya A. Shakri A. R. Yazdani S. S. Sharma M.. (2005). Mapping Binding Residues in the Plasmodium Vivax Domain That Binds Duffy Antigen During Red Cell Invasion. Mol. Microbiol. 55 (5), 1423–1434. doi: 10.1111/j.1365-2958.2005.04484.x 15720551

[B50] Haynes J. D. Dalton J. P. Klotz F. W. McGinniss M. H. Hadley T. J. Hudson D. E.. (1988). Receptor-Like Specificity of a Plasmodium Knowlesi Malarial Protein That Binds to Duffy Antigen Ligands on Erythrocytes. J. Exp. Med. 167 (6), 1873–1881. doi: 10.1084/jem.167.6.1873 2838562 PMC2189679

[B51] Herrera S. Corradin G. Arévalo-Herrera M.. (2007). An Update on the Search for a Plasmodium Vivax Vaccine. Trends Parasitol. 23 (3), 122–128. doi: 10.1016/j.pt.2007.01.008 17258937

[B52] Hester J. Chan E. R. Menard D. Mercereau-Puijalon O. Barnwell J. Zimmerman P. A.. (2013). *De Novo* Assembly of a Field Isolate Genome Reveals Novel Plasmodium Vivax Erythrocyte Invasion Genes. PLoS Negl. Trop. Dis. 7 (12), e2569. doi: 10.1371/journal.pntd.0002569 24340114 PMC3854868

[B53] Hisaeda H. Stowers A. W. Tsuboi T. Collins W. E. Sattabongkot J. S. Suwanabun N.. (2000). Antibodies to Malaria Vaccine Candidates Pvs25 and Pvs28 Completely Block the Ability of Plasmodium Vivax to Infect Mosquitoes. Infect. Immun. 68 (12), 6618–6623. doi: 10.1128/IAI.68.12.6618-6623.2000 11083773 PMC97758

[B54] Horuk R. Chitnis C. E. Darbonne W. C. Colby T. J. Rybicki A. Hadley T. J.. (1993). A Receptor for the Malarial Parasite Plasmodium Vivax: The Erythrocyte Chemokine Receptor. Science (New York N.Y.) 261 (5125), 1182–1184. doi: 10.1126/science.7689250 7689250

[B55] Hostetler J. B. Lo E. Kanjee U. Amaratunga C. Suon S. Sreng S.. (2016). Independent Origin and Global Distribution of Distinct Plasmodium Vivax Duffy Binding Protein Gene Duplications. PloS Negl. Trop. Dis. 10 (10), e0005091. doi: 10.1371/journal.pntd.0005091 27798646 PMC5087946

[B56] Howes R. E. Battle K. E. Mendis K. N. Smith D. L. Cibulskis R. E. Baird J. K.. (2016). Global Epidemiology of Plasmodium Vivax. Am. J. Trop. Med. Hyg. 95 (6 Suppl), 15–34. doi: 10.4269/ajtmh.16-0141 PMC5198891 27402513

[B57] Illingworth J. J. Alanine D. G. Brown R. Marshall J. M. Bartlett H. E. Silk S. E.. (2019). Functional Comparison of Blood-Stage Plasmodium Falciparum Malaria Vaccine Candidate Antigens. Front. Immunol. 10. doi: 10.3389/fimmu.2019.01254 PMC6558156 31214195

[B58] Imwong M. Snounou G. Pukrittayakamee S. Tanomsing N. Kim J. R. Nandy A.. (2007). Relapses of Plasmodium Vivax Infection Usually Result From Activation of Heterologous Hypnozoites. J. Infect. Dis. 195 (7), 927–933. doi: 10.1086/512241 17330781

[B59] Imwong M. Hanchana S. Malleret B. Rénia L. Day N. P. Dondorp A.. (2014). High-Throughput Ultrasensitive Molecular Techniques for Quantifying Low-Density Malaria Parasitemias. J. Clin. Microbiol. 52 (9), 3303–3309. doi: 10.1128/JCM.01057-14 24989601 PMC4313154

[B60] Ju H. L. Kang J. M. Moon S. U. Kim J. Y. Lee H. W. Lin K.. (2012). Genetic Polymorphism and Natural Selection of Duffy Binding Protein of Plasmodium Vivax Myanmar Isolates. Malaria J. 11, 60. doi: 10.1186/1475-2875-11-60 PMC3358247 22380592

[B61] Ju H. L. Kang J. M. Moon S. U. Bahk Y. Y. Cho P. Y. Sohn W. M.. (2013). Genetic Diversity and Natural Selection of Duffy Binding Protein of Plasmodium Vivax Korean Isolates. Acta Trop. 125 (1), 67–74. doi: 10.1016/j.actatropica.2012.09.016 23031445

[B62] Khatoon L.. (2010). Genetic Structure of Plasmodium Vivax and Plasmodium Falciparum in the Bannu District of Pakistan. Malaria J. 9, 112. doi: 10.1186/1475-2875-9-112 PMC2873525 20416089

[B63] Kho W. G. Chung J. Y. Sim E. J. Kim D. W. Chung W. C. (2001). Analysis of Polymorphic Regions of Plasmodium Vivax Duffy Binding Protein of Korean Isolates. Korean J. Parasitol. 39 (2), 143–150. doi: 10.3347/kjp.2001.39.2.143 11441501 PMC2721091

[B64] Kitchen S. F.. (1938). The Infection of Reticulocytes by Plasmodium Vivax. Am. J. Trop. Med. s1-18 (4), 347–359. doi: 10.4269/ajtmh.1938.s1-18.347

[B65] Kosaisavee V. Suwanarusk R. Chua A. Kyle D. E. Malleret B. Zhang R.. (2017). Strict Tropism for CD71+/CD234+ Human Reticulocytes Limits the Zoonotic Potential of Plasmodium Cynomolgi. Blood 130 (11), 1357–1363. doi: 10.1182/blood-2017-02-764787 28698207 PMC5600141

[B66] Krotoski W. A. Collins W. E. Bray R. S. Garnham P. C. Cogswell F. B. Gwadz R. W.. (1982). Demonstration of Hypnozoites in Sporozoite-Transmitted Plasmodium Vivax Infection. Am. J. Trop. Med. Hyg. 31 (6), 1291–1293. doi: 10.4269/ajtmh.1982.31.1291 6816080

[B67] Kwiatkowski D. P.. (2005). How Malaria has Affected the Human Genome and What Human Genetics can Teach Us About Malaria. Am. J. Hum. Genet. 77 (2), 171–192. doi: 10.1086/432519 16001361 PMC1224522

[B68] Lalremruata A. Magris M. Vivas-Martínez S. Koehler M. Esen M. Kempaiah P.. (2015). Natural Infection of Plasmodium Brasilianum in Humans: Man and Monkey Share Quartan Malaria Parasites in the Venezuelan Amazon. EBioMedicine 2 (9), 1186–1192. doi: 10.1016/j.ebiom.2015.07.033 26501116 PMC4588399

[B69] Laurens M. B.. (2020). RTS,S/AS01 Vaccine (Mosquirix™): An Overview. Hum. Vaccin. Immunother. 16 (3), 480–489. doi: 10.1080/21645515.2019.1669415 31545128 PMC7227679

[B70] Lo E. Hostetler J. B. Yewhalaw D. Pearson R. D. Hamid M. Gunalan K.. (2019). Frequent Expansion of Plasmodium Vivax Duffy Binding Protein in Ethiopia and its Epidemiological Significance. PloS Negl. Trop. Dis. 13 (9), e0007222. doi: 10.1371/journal.pntd.0007222 31509523 PMC6756552

[B71] Malleret B. Li A. Zhang R. Tan K. S. Suwanarusk R. Claser C.. (2015). Plasmodium Vivax: Restricted Tropism and Rapid Remodeling of CD71-Positive Reticulocytes. Blood 125 (8), 1314–1324. doi: 10.1182/blood-2014-08-596015 25414440 PMC4401350

[B72] Markus M. B. (2011). Malaria: Origin of the Term “Hypnozoite”. J. Hist. Biol. 44 (4), 781–786. doi: 10.1007/s10739-010-9239-3 20665090

[B73] Ménard D. Barnadas C. Bouchier C. Henry-Halldin C. Gray L. R. Ratsimbasoa A.. (2010). Plasmodium Vivax Clinical Malaria is Commonly Observed in Duffy-Negative Malagasy People. Proc. Natl. Acad. Sci. U.S.A. 107 (13), 5967–5971. doi: 10.1073/pnas.0912496107 20231434 PMC2851935

[B74] Menard D. Chan E. R. Benedet C. Ratsimbasoa A. Kim S. Chim P.. (2013). Whole Genome Sequencing of Field Isolates Reveals a Common Duplication of the Duffy Binding Protein Gene in Malagasy Plasmodium Vivax Strains. PloS Negl. Trop. Dis. 7 (11), e2489. doi: 10.1371/journal.pntd.0002489 24278487 PMC3836732

[B75] Miller L. H. Mason S. J. Clyde D. F. McGinniss M. H.. (1976). The Resistance Factor to Plasmodium Vivax in Blacks. The Duffy-Blood-Group Genotype, FyFy. N. Engl. J. Med. 295 (6), 302–304. doi: 10.1056/NEJM197608052950602 778616

[B76] Mittal P. Mishra S. Kar S. Pande V. Sinha A. Sharma A.. (2020). Global Distribution of Single Amino Acid Polymorphisms in Plasmodium Vivax Duffy-Binding-Like Domain and Implications for Vaccine Development Efforts. Open Biol. 10 (9), 200180. doi: 10.1098/rsob.200180 32993415 PMC7536081

[B77] Mohring F. Hart M. N. Rawlinson T. A. Henrici R. Charleston J. A. Diez Benavente E.. (2019). Rapid and Iterative Genome Editing in the Malaria Parasite *Plasmodium Knowlesi* Provides New Tools for *P. Vivax* Research. eLife 8, e45829. doi: 10.7554/eLife.45829 31205002 PMC6579517

[B78] Moon R. W. Hall J. Rangkuti F. Ho Y. S. Almond N. Mitchell G. H.. (2013). Adaptation of the Genetically Tractable Malaria Pathogen Plasmodium Knowlesi to Continuous Culture in Human Erythrocytes. Proc. Natl. Acad. Sci. U.S.A. 110 (2), 531–536. doi: 10.1073/pnas.1216457110 23267069 PMC3545754

[B79] Mueller I. Galinski M. R. Baird J. K. Carlton J. M. Kochar D. K. Alonso P. L.. (2009). Key Gaps in the Knowledge of Plasmodium Vivax, a Neglected Human Malaria Parasite. Lancet Infect. Dis. 9 (9), 555–566. doi: 10.1016/S1473-3099(09)70177-X 19695492

[B80] Mueller I. Shakri A. R. Chitnis C.E.. (2015). Development of Vaccines for Plasmodium Vivax Malaria. Vaccine 33 (52), 7489–7495. doi: 10.1016/j.vaccine.2015.09.060 26428453

[B81] Ndegwa D. N. Kundu P. Hostetler J. B. Marin-Menendez A. Sanderson T. Mwikali K.. (2021). Using Plasmodium Knowlesi as a Model for Screening Plasmodium Vivax Blood-Stage Malaria Vaccine Targets Reveals New Candidates. PLoS Pathog. 17 (7), e1008864. doi: 10.1371/journal.ppat.1008864 34197567 PMC8279373

[B82] Neafsey D. E. Galinsky K. Jiang R. H. Young L. Sykes S. M. Saif S.. (2012). The Malaria Parasite Plasmodium Vivax Exhibits Greater Genetic Diversity Than Plasmodium Falciparum. Nat. Genet. 44 (9), 1046–1050. doi: 10.1038/ng.2373 22863733 PMC3432710

[B83] Nicolete V. C. Frischmann S. Barbosa S. King C. L. Ferreira M. U.. (2016). Naturally Acquired Binding-Inhibitory Antibodies to Plasmodium Vivax Duffy Binding Protein and Clinical Immunity to Malaria in Rural Amazonians. J. Infect. Dis. 214 (10), 1539–1546. doi: 10.1093/infdis/jiw407 27578850 PMC5091372

[B84] Ntumngia F. B. Adams J. H.. (2012). Design and Immunogenicity of a Novel Synthetic Antigen Based on the Ligand Domain of the Plasmodium Vivax Duffy Binding Protein. Clin. Vaccine Immunol. 19 (1), 30–36. doi: 10.1128/CVI.05466-11 22116684 PMC3255949

[B85] Ntumngia F. B. Schloegel J. Barnes S. J. McHenry A. M. Singh S. King C. L.. (2012). Conserved and Variant Epitopes of Plasmodium Vivax Duffy Binding Protein as Targets of Inhibitory Monoclonal Antibodies. Infect. Immun. 80 (3), 1203–1208. doi: 10.1128/IAI.05924-11 22215740 PMC3294652

[B86] Ntumngia F. B. Schloegel J. McHenry A. M. Barnes S. J. George M. T.. (2013). Immunogenicity of Single Versus Mixed Allele Vaccines of Plasmodium Vivax Duffy Binding Protein Region II. Vaccine 31 (40), 4382–4388. doi: 10.1016/j.vaccine.2013.07.002 23916294 PMC4497540

[B87] Ntumngia F. B. Thomson-Luque R. Torres L. Gunalan K. Carvalho L. H. Adams J. H.. (2016). A Novel Erythrocyte Binding Protein of Plasmodium Vivax Suggests an Alternate Invasion Pathway Into Duffy-Positive Reticulocytes. mBio 7 (4), e01261–e01216. doi: 10.1128/mBio.01261-16 27555313 PMC4999553

[B88] Ntumngia F. B. Thomson-Luque R. Galusic S. Frato G. Frischmann S. Peabody D. S.. (2018). Identification and Immunological Characterization of the Ligand Domain of Plasmodium Vivax Reticulocyte Binding Protein 1a. J. Infect. Dis. 218 (7), 1110–1118. doi: 10.1093/infdis/jiy273 29741629 PMC6107737

[B89] Ogun S. A. Tewari R. Otto T. D. Howell S. A. Knuepfer E. Cunningham D. A.. (2011). Targeted Disruption of Py235ebp-1: Invasion of Erythrocytes by Plasmodium Yoelii Using an Alternative Py235 Erythrocyte Binding Protein. PLoS Pathog. 7 (2), e1001288. doi: 10.1371/journal.ppat.1001288 21379566 PMC3040676

[B90] Okenu D. M. Meyer E. V. Puckett T. C. Rosas-Acosta G. Barnwell J. W. Galinski M. R.. (2005). The Reticulocyte Binding Proteins of Plasmodium Cynomolgi: A Model System for Studies of P. Vivax. Mol. Biochem. Parasitol. 143 (1), 116–120. doi: 10.1016/j.molbiopara.2005.04.010 15990180

[B91] Parzych E. M. Miura K. Ramanathan A. Long C. A. Burns J. M.. (2017). Evaluation of a Plasmodium-Specific Carrier Protein To Enhance Production of Recombinant Pfs25, a Leading Transmission-Blocking Vaccine Candidate. Infect. Immun. 86 (1), e00486–e00417. doi: 10.1128/IAI.00486-17 28993460 PMC5736822

[B92] Patarroyo M. A. Molina-Franky J. Gómez M. Arévalo-Pinzón G. Patarroyo M. E.. (2020). Hotspots in Plasmodium and RBC Receptor-Ligand Interactions: Key Pieces for Inhibiting Malarial Parasite Invasion. Int. J. Mol. Sci. 21 (13), 4729. doi: 10.3390/ijms21134729 PMC7370042 32630804

[B93] Payne R. O. Silk S. E. Elias S. C. Milne K. H. Rawlinson T. A. Llewellyn D.. (2017). Human Vaccination Against Plasmodium Vivax Duffy-Binding Protein Induces Strain-Transcending Antibodies. JCI Insight 2 (12), e93683. doi: 10.1172/jci.insight.93683 PMC5470884 28614791

[B94] Premaratne P. H. Aravinda B. R. Escalante A. A. Udagama P.V.. (2011). Genetic Diversity of Plasmodium Vivax Duffy Binding Protein II (PvDBPII) Under Unstable Transmission and Low Intensity Malaria in Sri Lanka. Infect. Genet. Evol. 11 (6), 1327–1339. doi: 10.1016/j.meegid.2011.04.023 21554998

[B95] Price R. N.. (2014). Global Extent of Chloroquine-Resistant Plasmodium Vivax: A Systematic Review and Meta-Analysis. Lancet Infect. Dis. 14 (10), 982–991. doi: 10.1016/S1473-3099(14)70855-2 25213732 PMC4178238

[B96] Price R. N. Tjitra E. Guerra C. A. Yeung S. White N. J. Anstey N. M.. (2007). Vivax Malaria: Neglected and Not Benign. Am. J. Trop. Med. Hyg. 77 (6 Suppl), 79–87. 18165478 PMC2653940

[B97] Price R. N. Douglas N. M. Anstey N. M.. (2009). New Developments in Plasmodium Vivax Malaria: Severe Disease and the Rise of Chloroquine Resistance. Curr. Opin. Infect. Dis. 22 (5), 430–435. doi: 10.1097/QCO.0b013e32832f14c1 19571748

[B98] Qian F. Wu Y. Muratova O. Zhou H. Dobrescu G. Duggan P.. (2007). Conjugating Recombinant Proteins to Pseudomonas Aeruginosa ExoProtein A: A Strategy for Enhancing Immunogenicity of Malaria Vaccine Candidates. Vaccine 25 (20), 3923–3933. doi: 10.1016/j.vaccine.2007.02.073 17428587 PMC1940062

[B99] Radtke A. J. Anderson C. F. Riteau N. Rausch K. Scaria P. Kelnhofer E. R.. (2017). Adjuvant and Carrier Protein-Dependent T-Cell Priming Promotes a Robust Antibody Response Against the Plasmodium Falciparum Pfs25 Vaccine Candidate. Sci. Rep. 7, 40312. doi: 10.1038/srep40312 28091576 PMC5238395

[B100] Ranjan A. Chitnis C. E.. (1999). Mapping Regions Containing Binding Residues Within Functional Domains of Plasmodium Vivax and Plasmodium Knowlesi Erythrocyte-Binding Proteins. Proc. Natl. Acad. Sci. U.S.A. 96 (24), 14067–14072. doi: 10.1073/pnas.96.24.14067 10570199 PMC24191

[B101] Rawlinson T. A. Barber N. M. Mohring F. Cho J. S. Kosaisavee V. Gérard S. F.. (2019). Structural Basis for Inhibition of Plasmodium Vivax Invasion by a Broadly Neutralizing Vaccine-Induced Human Antibody. Nat. Microbiol. 4 (9), 1497–1507. doi: 10.1038/s41564-019-0462-1 31133755 PMC6711757

[B102] Registration T. Tables of Malaria Vaccine Projects Globally. Available at: http://www.who.int/immunization/research/development/Rainbow tables/en/.

[B103] Rieckmann K. H. Davis D. R. Hutton D. C.. (1989). Plasmodium Vivax Resistance to Chloroquine? Lancet (Lond. Engl.) 2 (8673), 1183–1184. doi: 10.1016/s0140-6736(89)91792-3 2572903

[B104] Roesch C. Popovici J. Bin S. Run V. Kim S. Ramboarina S.. (2018). Genetic Diversity in Two Plasmodium Vivax Protein Ligands for Reticulocyte Invasion. PLoS Negl. Trop. Dis. 12 (10), e0006555. doi: 10.1371/journal.pntd.0006555 30346980 PMC6211765

[B105] RTS,S Clinical Trials Partnership. (2014). Efficacy and Safety of the RTS,S/AS01 Malaria Vaccine During 18 Months After Vaccination: A Phase 3 Randomized, Controlled Trial in Children and Young Infants at 11 African Sites. PLoS Med. 11 (7), e1001685. doi: 10.1371/journal.pmed.1001685 25072396 PMC4114488

[B106] Ryan J. R. Stoute J. A. Amon J. Dunton R. F. Mtalib R. Koros J.. (2006). Evidence for Transmission of Plasmodium Vivax Among a Duffy Antigen Negative Population in Western Kenya. Am. J. Trop. Med. Hyg. 75 (4), 575–581. doi: 10.4269/ajtmh.2006.75.575 17038676

[B107] Sanger R. Race R. R. Jack J.. (1955). The Duffy Blood Groups of New York Negroes: The Phenotype Fy (a-B-). Br. J. Haematol. 1 (4), 370–374. doi: 10.1111/j.1365-2141.1955.tb05523.x 13269673

[B108] Sauerwein R. W. Bousema T.. (2015). Transmission Blocking Malaria Vaccines: Assays and Candidates in Clinical Development. Vaccine 33 (52), 7476–7482. doi: 10.1016/j.vaccine.2015.08.073 26409813

[B109] Schunk M. Kumma W. P. Miranda I. B. Osman M. E. Roewer S. Alano A.. (2006). High Prevalence of Drug-Resistance Mutations in Plasmodium Falciparum and Plasmodium Vivax in Southern Ethiopia. Malar. J. 5, 54. doi: 10.1186/1475-2875-5-54 16817953 PMC1524791

[B110] Singh S. K. Singh A. P. Pandey S. Yazdani S. S. Chitnis C. E. Sharma A.. (2003). Definition of Structural Elements in Plasmodium Vivax and P. Knowlesi Duffy-Binding Domains Necessary for Erythrocyte Invasion. Biochem. J. 374 (Pt 1), 193–198. doi: 10.1042/BJ20030622 12775212 PMC1223586

[B111] Singh S. K. Hora R. Belrhali H. Chitnis C. E. Sharma A.. (2006). Structural Basis for Duffy Recognition by the Malaria Parasite Duffy-Binding-Like Domain. Nature 439 (7077), 741–744. doi: 10.1038/nature04443 16372020

[B112] Singh K. Mukherjee P. Shakri A. R. Singh A. Pandey G. Bakshi M.. (2018). Malaria Vaccine Candidate Based on Duffy-Binding Protein Elicits Strain Transcending Functional Antibodies in a Phase I Trial. NPJ Vaccines 3, 48. doi: 10.1038/s41541-018-0083-3 30302285 PMC6162314

[B113] Sousa T. N. Cerávolo I. P. Fernandes Fontes C. J. Couto A. Carvalho L. H. Brito C. F.. (2006). The Pattern of Major Polymorphisms in the Duffy Binding Protein Ligand Domain Among Plasmodium Vivax Isolates From the Brazilian Amazon Area. Mol. Biochem. Parasitol. 146 (2), 251–254. doi: 10.1016/j.molbiopara.2005.11.006 16384615

[B114] Su X. Z.. (2019). Plasmodium Genomics and Genetics: New Insights Into Malaria Pathogenesis, Drug Resistance, Epidemiology, and Evolution. Clin. Microbiol. Rev. 32 (4), e00019–e00019. doi: 10.1128/CMR.00019-19 31366610 PMC6750138

[B115] Sutherland C. J.. (2010). Two Nonrecombining Sympatric Forms of the Human Malaria Parasite Plasmodium Ovale Occur Globally. J. Infect. Dis. United States 201 (10), 1544–1550. doi: 10.1086/652240 20380562

[B116] Tachibana M. Suwanabun N. Kaneko O. Iriko H. Otsuki H. Sattabongkot J.. (2015). Plasmodium Vivax Gametocyte Proteins, Pvs48/45 and Pvs47, Induce Transmission-Reducing Antibodies by DNA Immunization. Vaccine 33 (16), 1901–1908. doi: 10.1016/j.vaccine.2015.03.008 25765968

[B117] Ta T. H. Hisam S. Lanza M. Jiram A. I. Ismail N. Rubio J. M.. (2014). First Case of a Naturally Acquired Human Infection With Plasmodium Cynomolgi. Malaria J. 13 (1), 1–7. doi: 10.1186/1475-2875-13-68 PMC3937822 24564912

[B118] Tanner M. Greenwood B. Whitty C. J. Ansah E. K. Price R. N. Dondorp A. M.. (2015). Malaria Eradication and Elimination: Views on How to Translate a Vision Into Reality. BMC Med. 13, 167. doi: 10.1186/s12916-015-0384-6 26208740 PMC4514994

[B119] Tsuboi T. Kappe S. H. al-Yaman F. Prickett M. D. Alpers M. Adams J. H.. (1994). Natural Variation Within the Principal Adhesion Domain of the Plasmodium Vivax Duffy Binding Protein. Infect. Immun. 62 (12), 5581–5586. doi: 10.1128/iai.62.12.5581-5586.1994 7960140 PMC303305

[B120] Tsuboi T. Kaslow D. C. Gozar M. M. Tachibana M. Cao Y. M. Torii M.. (1998). Sequence Polymorphism in Two Novel Plasmodium Vivax Ookinete Surface Proteins, Pvs25 and Pvs28, That are Malaria Transmission-Blocking Vaccine Candidates. Mol. Med. (Cambridge Mass.) 4 (12), 772–782. doi: 10.1007/BF03401770 9990863 PMC2230397

[B121] Urusova D. Carias L. Huang Y. Nicolete V. C. Popovici J. Roesch C.. (2019). Structural Basis for Neutralization of Plasmodium Vivax by Naturally Acquired Human Antibodies That Target DBP. Nat. Microbiol. 4 (9), 1486–1496. doi: 10.1038/s41564-019-0461-2 31133752 PMC6707876

[B122] Valencia S. H. Rodríguez D. C. Acero D. L. Ocampo V. Arévalo-Herrera M.. (2011). Platform for Plasmodium Vivax Vaccine Discovery and Development. Mem. Inst. Oswaldo Cruz 106 Suppl 1 (Suppl 1), 179–192. doi: 10.1590/s0074-02762011000900023 21881773 PMC4832982

[B123] VanBuskirk K. M. Cole-Tobian J. L. Baisor M. Sevova E. S. Bockarie M. King C. L.. (2004a). Antigenic Drift in the Ligand Domain of Plasmodium Vivax Duffy Binding Protein Confers Resistance to Inhibitory Antibodies. J. Infect. Dis. 190 (9), 1556–1562. doi: 10.1086/424852 15478059

[B124] VanBuskirk K. M. Sevova E. Adams J. H. (2004b). Conserved Residues in the Plasmodium Vivax Duffy-Binding Protein Ligand Domain are Critical for Erythrocyte Receptor Recognition. Proc. Natl. Acad. Sci. U.S.A. 101 (44), 15754–15759. doi: 10.1073/pnas.0405421101 15498870 PMC524844

[B125] Vicentin E. C. Françoso K. S. Rocha M. V. Iourtov D. Dos Santos F. L. Kubrusly F. S.. (2014). Invasion-Inhibitory Antibodies Elicited by Immunization With Plasmodium Vivax Apical Membrane Antigen-1 Expressed in Pichia Pastoris Yeast. Infect. Immun. 82 (3), 1296–1307. doi: 10.1128/IAI.01169-13 24379279 PMC3958008

[B126] White N. J.. (2021). Anti-Malarial Drug Effects on Parasite Dynamics in Vivax Malaria. Malaria J. 20 (1), 161. doi: 10.1186/s12936-021-03700-7 PMC7981980 33743731

[B127] Winter D. J. Pacheco M. A. Vallejo A. F. Schwartz R. S. Arevalo-Herrera M. Herrera S.. (2015). Whole Genome Sequencing of Field Isolates Reveals Extensive Genetic Diversity in Plasmodium Vivax From Colombia. PLoS Negl. Trop. Dis. 9 (12), e0004252. doi: 10.1371/journal.pntd.0004252 26709695 PMC4692395

[B128] Woldearegai T. G. Kremsner P. G. Kun J. F. Mordmüller B.. (2013). Plasmodium Vivax Malaria in Duffy-Negative Individuals From Ethiopia. Trans. R. Soc. Trop. Med. Hyg. 107 (5), 328–331. doi: 10.1093/trstmh/trt016 23584375

[B129] World Health Organisation. (2021). World Malaria Report 2021 (Geneva, Switzerland: World Health Organization).

[B130] World Health Organization. (2020). World Malaria Report 2020 (Geneva, Switzerland: World Health Organization).

[B131] Wu Y. Ellis R. D. Shaffer D. Fontes E. Malkin E. M. Mahanty S.. (2008). Phase 1 Trial of Malaria Transmission Blocking Vaccine Candidates Pfs25 and Pvs25 Formulated With Montanide ISA 51. PLoS One 3 (7), e2636. doi: 10.1371/journal.pone.0002636 18612426 PMC2440546

[B132] Xainli J. Adams J. H. King C. L.. (2000). The Erythrocyte Binding Motif of Plasmodium Vivax Duffy Binding Protein is Highly Polymorphic and Functionally Conserved in Isolates From Papua New Guinea. Mol. Biochem. Parasitol. 111 (2), 253–260. doi: 10.1016/s0166-6851(00)00315-7 11163434

[B133] Yogavel M. Chhibber-Goel J. Jamwal A. Gupta S. Sharma A.. (2018). Engagement Rules That Underpin DBL-DARC Interactions for Ingress of Plasmodium Knowlesi and Plasmodium Vivax Into Human Erythrocytes. Front. Mol. Biosci. 5. doi: 10.3389/fmolb.2018.00078 PMC6120517 30211170

